# Viral Retasking of hBre1/RNF20 to Recruit hPaf1 for Transcriptional Activation

**DOI:** 10.1371/journal.ppat.1003411

**Published:** 2013-06-13

**Authors:** Gregory J. Fonseca, Michael J. Cohen, Anthony C. Nichols, John W. Barrett, Joe S. Mymryk

**Affiliations:** 1 Department of Microbiology & Immunology, University of Western Ontario, London, Ontario, Canada; 2 Department of Otolaryngology-Head and Neck Surgery, University of Western Ontario, London, Ontario, Canada; 3 Department of Oncology, University of Western Ontario, London, Ontario, Canada; 4 London Regional Cancer Program and Lawson Health Research Institute, London, Ontario, Canada; Salk Institute, United States of America

## Abstract

Upon infection, human adenovirus (HAdV) must activate the expression of its early genes to reprogram the cellular environment to support virus replication. This activation is orchestrated in large part by the first HAdV gene expressed during infection, early region 1A (E1A). E1A binds and appropriates components of the cellular transcriptional machinery to modulate cellular gene transcription and activate viral early genes transcription. Previously, we identified hBre1/RNF20 as a target for E1A. The interaction between E1A and hBre1 antagonizes the innate antiviral response by blocking H2B monoubiquitination, a chromatin modification necessary for the interferon (IFN) response. Here, we describe a second distinct role for the interaction of E1A with hBre1 in transcriptional activation of HAdV early genes. Furthermore, we show that E1A changes the function of hBre1 from a ubiquitin ligase involved in substrate selection to a scaffold which recruits hPaf1 as a means to stimulate transcription and transcription-coupled histone modifications. By using hBre1 to recruit hPaf1, E1A is able to optimally activate viral early transcription and begin the cycle of viral replication. The ability of E1A to target hBre1 to simultaneously repress cellular IFN dependent transcription while activating viral transcription, represents an elegant example of the incredible economy of action accomplished by a viral regulatory protein through a single protein interaction.

## Author Summary

Adenovirus typically infects the upper respiratory tract and is one of the viral groups that causes the common cold. The virus itself is unable to reproduce on its own and so must use the infected host cell to produce more virus. Adenovirus reprograms the infected cell using the early gene products. The transcription of these viral proteins is activated by the product of the first viral gene produced during adenoviral infection, early region 1A (E1A). Two regions of the E1A protein are responsible for producing the adenovirus early proteins, the N-terminal and conserved region 3 (CR3) regions. Although much is known about the mechanism by which CR3 activates transcription, less is known about the way by which the N-terminal region functions. Here, we describe how the interaction of the N-terminal region of E1A with the hBre1/RNF20 ubiquitin ligase is involved in viral early gene activation. E1A uses hBre1 as a scaffold to recruit the transcriptional regulator hPaf1 to the viral E3 and E4 early gene promoters. This is necessary for several histone post-translational modifications, efficient activation of E3 and E4 expression, and this ultimately contributes to viral replication.

## Introduction

Viruses are obligate intracellular pathogens as they require cellular machinery to replicate. Indeed, viruses often subvert the functions of cellular machinery to support their life cycle. Human adenovirus (HAdV) is no exception, and during infection must appropriate the host cellular transcriptional apparatus to begin transcription of the viral genes necessary to reprogram the cellular environment [1], [2]. This is done in large part by the viral products of Early Region 1A (E1A), the first gene transcribed after infection. The E1A proteins bind and redirect the activity of transcriptional regulators to initiate transcription of the HAdV early genes [2], [3]. The HAdV 5 E1A mRNA has five splice variants. The two largest isoforms, 13S and 12S, encode 289 and 243 residue (R) proteins, respectively. These proteins predominate at the early stages of virus infection. Sequence alignment of E1A from a variety of HAdVs shows four regions of conservation, and have been designated CR1-4 [4]. The 289R and 243R E1A proteins of HAdV 5 are identical except for the presence of an additional 46 amino acid sequence within the 289R [5]. This unique 46 amino acid region encompasses CR3 [6]. Both the CR3 region and N-terminal 82 residues of E1A are sufficient to activate transcription when fused to a heterologous DNA binding domain [7], [8]. Although each region can separately recruit a plethora of transcriptional activators [6], [8]–[12], they function together to recruit cellular transcriptional complexes for the activation of viral transcription [5], [6], [13], [14]. CR3, specifically, activates transcription through interactions with the mediator complex component Med23 (mediator complex subunit 23) [9], [15], [16]. CR3 activity is further modulated by pCaf (CREBBP-associated factor), Gcn5 (general control of amino-acid synthesis, yeast, homolog), p300 (E1A binding protein p300), BS69 (bone morphogenetic protein receptor-associated molecule 1) and Sug1 (26S proteasome AAA-ATPase subunit RPT6) [11], [15], [17]–[19]. Likewise, the N-terminus of E1A interacts with transcriptional activators, such as p300, CBP (CREB-binding protein), p400 (E1A binding protein p400), pCaf, TBP (TATA binding protein), and TRAAP (transformation/transcription domain-associated protein) [1]. Although there exists a large body of research focusing on the role that CR3 plays in virus transcription, the requirement for the N-terminus, which is conserved in both the 289R and 243R E1A proteins, and the mechanisms through which it cooperates with CR3 to activate viral transcription, are poorly understood.

Previously, we identified a novel interaction between the N-terminus of HAdV 5 E1A and hBre1(human BREfeldin A sensitivity)/RNF20 (Ring finger protein 20) [20]. hBre1 is a cellular ubiquitin ligase, which functions in concert with the accessory factor RNF40 (Ring finger protein 40) and the Ube2b (Ubiquitin-conjugating enzyme E2B) ubiquitin conjugase to monoubiquitinate histone 2B (H2B) [21], [22]. The monoubiquitination of H2B (H2B-ub) is an epigenetic mark of genes that are highly transcriptionally active [23]. H2B-ub is a precursor to other activation marks, such as the trimethylation of histone 3 lysines 4 and 79, which are promoted by the interaction of the hBre1 complex with the hPaf1 (human RNA polymerase II associated factor 1) complex [24], [25]. During infection, E1A binds to hBre1 and blocks the interaction between hBre1 and the catalytic subunit Ube2b. This specifically antagonizes the ability of the hBre1 complex to monoubiquitinate H2B at interferon (IFN) responsive genes [20]. In this way, the interaction of E1A with hBre1 blocks transcriptional activation of IFN responsive genes and this contributes to inhibition of the cellular innate immune response to HAdV infection via an epigenetic mechanism [20].

Here, we investigate whether the interaction of HAdV 5 E1A with hBre1 influences transcription from the HAdV genome. We have found that the interaction of E1A with hBre1 contributes to the activation of early viral gene expression. Specifically, E1A utilizes hBre1 as a scaffolding protein to recruit the hPaf1 complex to HAdV early genes. This retasking of the hPaf1 complex, which is known to promote RNA polymerase II transcription elongation and transcription-coupled histone modifications, contributes to E1A mediated activation of HAdV early gene expression. Thus, in addition to antagonizing the ability of hBre1 to function as an E3 ubiquitin ligase involved in transcriptional activation of the innate immune response, the interaction of E1A with hBre1 serves a second distinct purpose as a novel means of enhancing viral gene transcription by recruiting the hPaf1 complex.

## Results

### hBre1 contributes to Gal4 mediated transcriptional activation by the N-terminus

**Figure 1:**
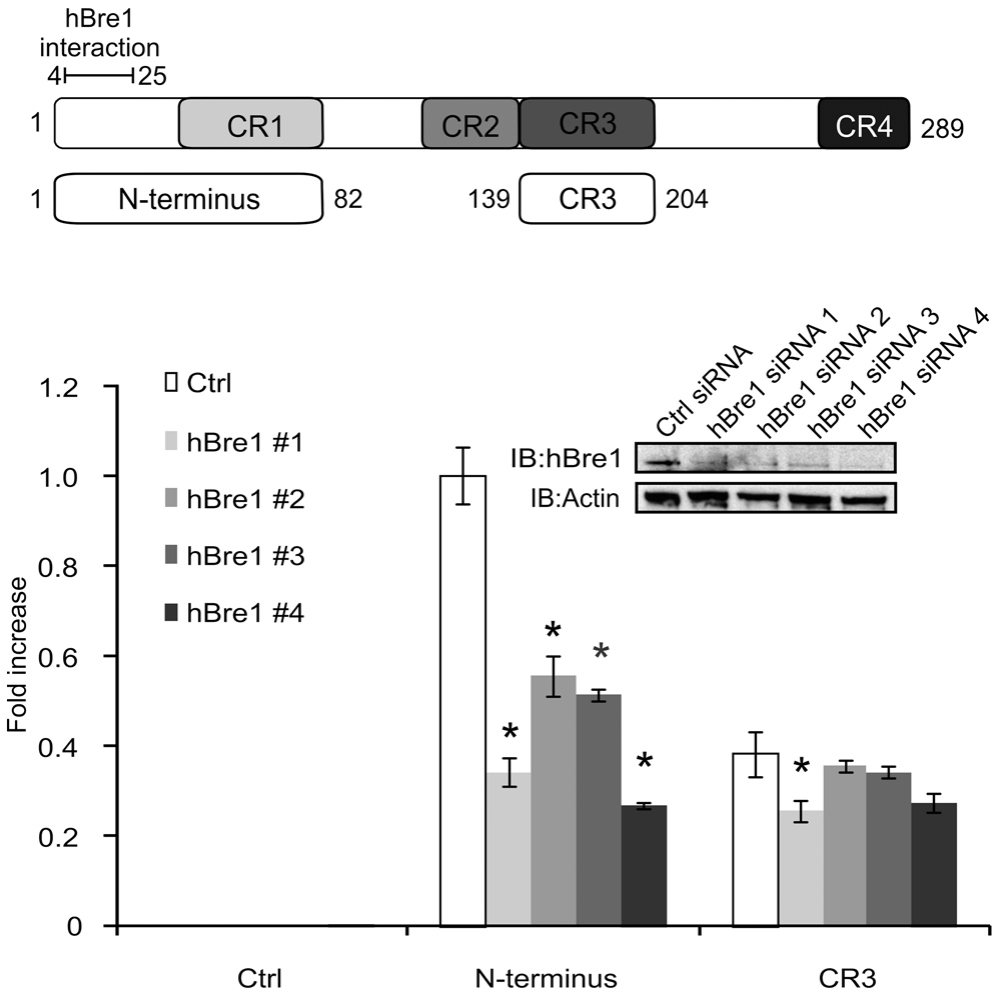
The N-terminus of E1A specifically requires hBre1 for full activation of transcription. U-2 OS cells were transfected with a negative control siRNA or 1 of 4 siRNAs specific for hBre1. Cells were then transfected with a constitutive β-galactosidase reporter, a Gal4 responsive luciferase reporter and a vector expressing the Gal4 DNA binding domain (DBD) alone, the Gal4 DBD fused to the N-terminus of E1A, or the Gal4 DBD fused to E1A CR3. Luciferase activity was measured. Results were normalized to β-galactosidase activity and siRNA treated groups were set as a fold change to the Gal4 only transfected counterpart. A statistically significant decrease from control siRNA treatment is indicated (* P<0.01). n = 3.

We previously showed that the N-terminus of E1A interacts with the cellular ubiquitin ligase hBre1 [20]. To determine if hBre1 influences transcriptional activation by the N-terminus of E1A, human U-2 OS osteosarcoma cells were treated with either a control (Ctrl) siRNA or one of 4 hBre1 specific siRNAs. Cells were then transfected with a constitutive β-galactosidase reporter, a Gal4 responsive luciferase reporter and a vector expressing the Gal4 DNA binding domain (DBD) alone or Gal4 DBD fused to the N-terminus of E1A. As an additional control, cells were similarly transfected with a vector expressing the Gal4 DBD fused to E1A CR3. E1A CR3 also functions as a strong transcriptional activation domain, but does not interact with hBre1. Luciferase activities were measured and results were normalized to β-galactosidase activity. The effects of siRNA treatment were calculated as a relative fold change with respect to cells transfected with a vector expressing the Gal4 DBD alone. Treatment with each of the 4 hBre1 specific siRNAs resulted in decreased hBre1 expression, which was accompanied by a decrease in E1A N-terminal dependent transcriptional activation (Figure 1). Based on this result, we used hBre1 siRNA 3 for further experiments. In contrast to its effects on transactivation by the N-terminus of E1A, knockdown of hBre1 had only modest effects on transcriptional activation by E1A CR3. These data suggest that hBre1 is involved transcriptional activation mediated by the N-terminus of E1A, but not by CR3.

### E1A requires hBre1 and likely p300/CBP to fully activate transcription of viral early genes

**Figure 2:**
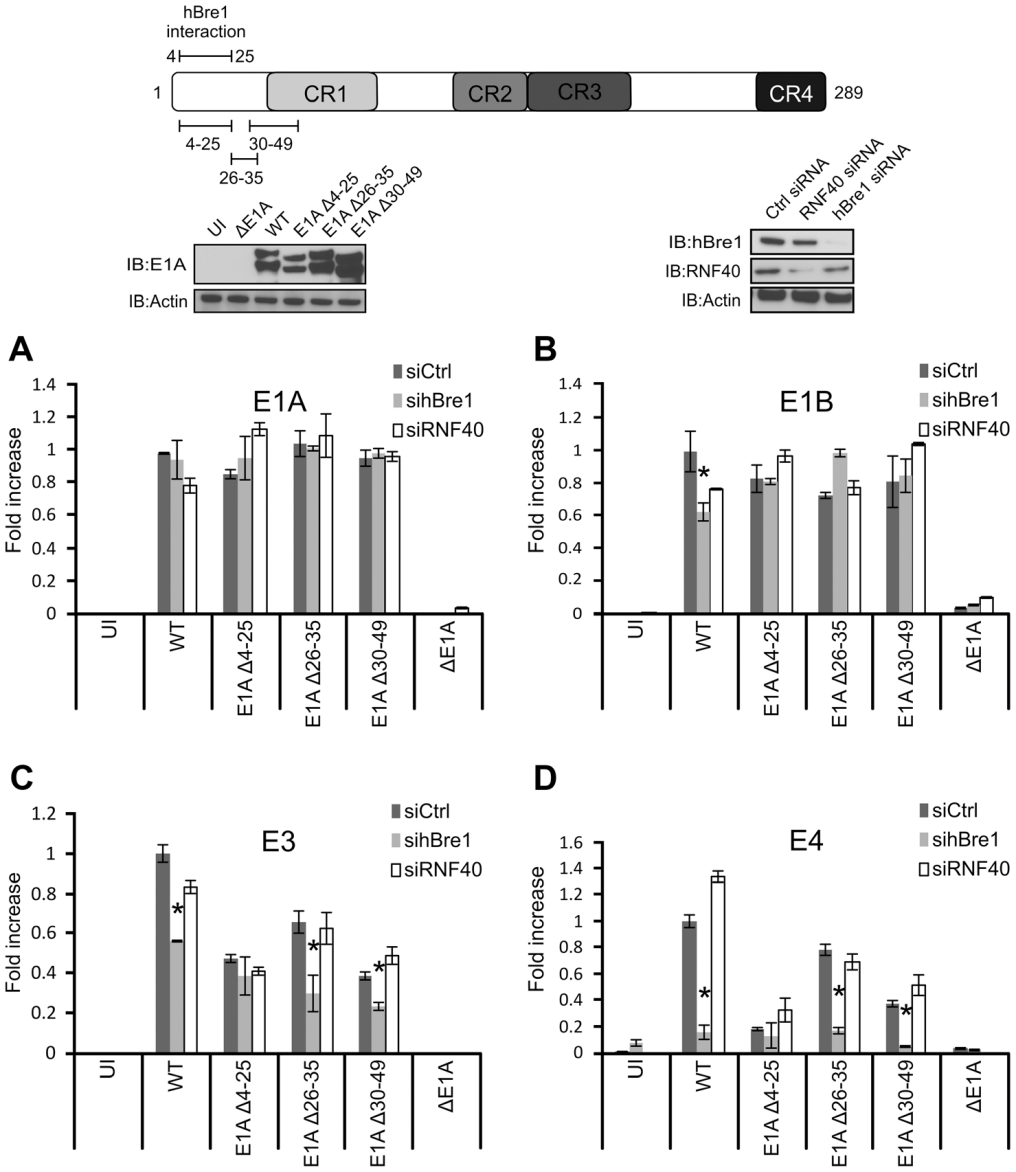
hBre1 contributes to E1A mediated activation of E3 and E4 expression during infection with HAdV. A549 cells were treated with Ctrl siRNA, siRNA specific to RNF40 or siRNA specific for hBre1 (Shown in the inset panel) and infected at an MOI of 5 with WT HAdV, or a series of HAdV containing the indicated deletions within the N-terminus of E1A (Shown in the inset panel). RT-qPCR was performed with a panel of HAdV early genes, normalized to GAPDH and fold change to uninfected Ctrl siRNA treated cells was plotted. Results for the HAdV E1A (A), E1B (B), E3 (C), and E4 (D) transcription units are shown. A statistically significant decrease from siCtrl is indicated (* P<0.01). n = 3.

To determine if the hBre1 complex is required for E1A dependent activation of viral early gene expression, human A549 lung epithelial cells were treated with Ctrl siRNA or siRNA specific to either hBre1 or the hBre1 complex member RNF40 and were then infected with either wildtype (WT; dl309) HAdV or a series of HAdV E1A deletion mutants at a multiplicity of infection (MOI) of 5. These viruses express both the 289R and 243R E1A proteins. cDNA was prepared from cells collected 20 hours post infection (h.p.i). Under these conditions of infection, this time point precedes the onset of viral late gene transcription and expression, as well as amplification of the viral genome (Figure S1 in Text S1). The expression of a panel of HAdV early genes known to be activated by E1A was determined by quantitative real time PCR. Knockdown of hBre1 did not consistently reduce expression from the viral E1A or E1B transcription units over the panel of viruses (Figure 2A,B). However, mRNA levels were significantly decreased from both the E3 and E4 transcription units in cells treated with hBre1 specific siRNA (Figure 2C and D). In contrast, treatment with siRNA specific for the RNF40 component of the hBre1 complex did not significantly decrease mRNA levels from any of the HAdV early genes (Figure 2, all panels). This result was unexpected, as RNF40 is essential for monoubiquitination of H2B by the hBre1 complex [21]. Infection with a virus containing a deletion of E1A that is unable to bind hBre1 (E1A Δ4–25), also showed a reduction in transcription of E3 and E4, but not E1A and E1B (Figure 2). Interestingly, the decreased early gene expression observed with this mutant was not further exacerbated by knockdown of hBre1. This suggested that the inability of the E1A Δ4–25 mutant to bind hBre1 and the knockdown of hBre1 might have redundant effects on E1A dependent transcription of E3 and E4 (Figure 2C and D).

A reduction in the transcriptional activation of E3 and E4 was also observed during infection with virus containing E1A deletions in adjacent regions not required for interaction with hBre1 (E1A Δ26–35, E1A Δ30–49; Figure 2C and D). In contrast to E1A Δ4–25, transcription of E3 and E4 during infection with these mutants was further decreased by knockdown of hBre1 (Figure 2C and D). Taken together, these results suggest that, while hBre1 is specifically targeted by E1A to enhance transcriptional activation of E3 and E4, other factors bound at adjacent regions of E1A also contribute to full transcriptional activation.

It should be noted that residues 4–25 of E1A are also required for interaction with p300 and CBP as well as hBre1 [20], [26], [27]. p300/CBP may also play a role in E1A-dependent transactivation as the mutant lacking residues 30–49, which are also required for p300/CBP interaction [26], [27] but not hBre1 binding [20], also shows a reduction of E3 and E4 expression (Figure 2C, 2D).

### hBre1 is recruited to HAdV early genes by E1A, but this does not lead to H2B monoubiquitination

**Figure 3:**
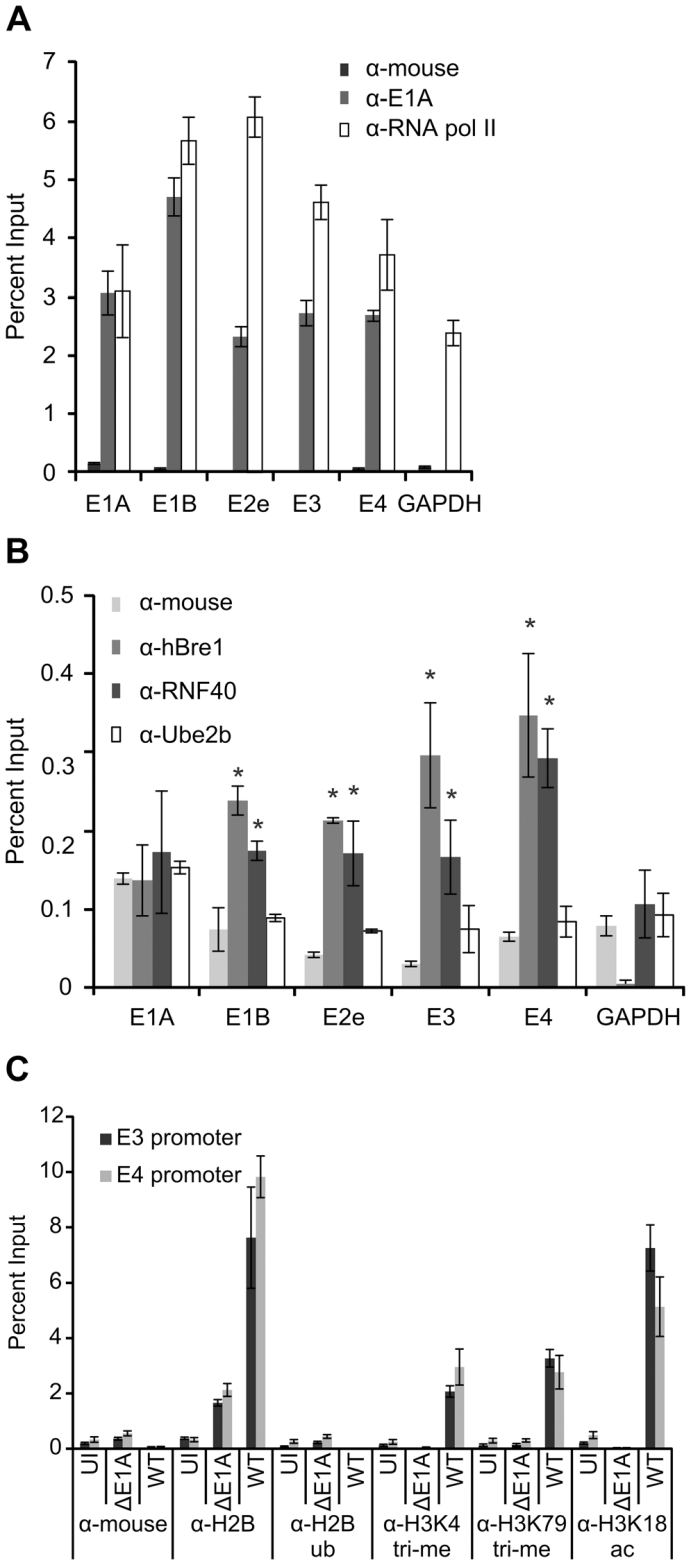
E1A recruits hBre1 to HAdV early gene promoters, but HAdV chromatin is not monoubiquitinated by hBre1. A549 cells were infected with WT HAdV or ΔE1A HAdV for 20 hours. Chromatin immunoprecipitation (ChIP) was then performed with antibodies specific for the indicated proteins. DNA was probed via qRT-PCR for the presence of HAdV early gene promoters. Data was normalized to ΔE1A HAdV and a non-specific antibody control. Occupancy by RNA pol II and E1A (A), hBre1 complex members (B), and the indicated histone post-translational modifications (C) at HAdV early gene promoters are shown. In B, a statistically significant increase from mouse antibody ChIP is indicated (* P<0.01). n = 3.

As hBre1, but not RNF40 is required for maximal E1A dependent activation of E3 and E4 expression (Figure 2), we next determined whether hBre1 complex members or H2B-ub could be detected on the E3 and E4 promoters. A549 cells were infected with WT HAdV or a HAdV containing a deletion of E1A (ΔE1A) at an MOI of 5. Chromatin immunoprecipitation (ChIP) was then performed using an antibody control, E1A specific antibody, RNA polymerase II (RNA pol II) specific antibody, and antibodies specific to hBre1 complex members hBre1, RNF40, and Ube2b. As expected [28], E1A was present at all HAdV early gene promoters and this correlated with the transcriptional status as determined by occupancy of RNA pol II (Figure 3A). E1A was not detected on the cellular GAPDH (glyceraldehyde-3-phosphate dehydrogenase) promoter, as has been previously published [20], [29]. Similarly, hBre1 and RNF40 were specifically associated with the promoters of the HAdV early genes E2e, E3, and E4, but not with E1A or E1B (Figure 3B and S2 in Text S1). In contrast, Ube2b, the catalytic member of the hBre1 complex involved in H2B monoubiquitination, was not recruited to the HAdV genome. Given that RNF40 is not required for transcriptional activation of HAdV early genes, and Ube2b is not present on their promoters, it seems unlikely that the hBre1 complex could perform its best characterized function, the monoubiquitination of H2B. This was confirmed by ChIP analysis, which demonstrated that H2B-ub was not present on viral chromatin at the E3 and E4 promoters (Figure 3C). Despite an absence of H2B-ub, marks of transcriptional activation such as H3K18 acetylation, and H3K4 and K79 trimethylation were detected by ChIP on the E3 and E4 promoters (Figure 3C), which is consistent with the presence of RNA pol II (Figure 3A).

### hPaf1 contributes to transcriptional activation by the N-terminus of E1A, but not by CR3

**Figure 4:**
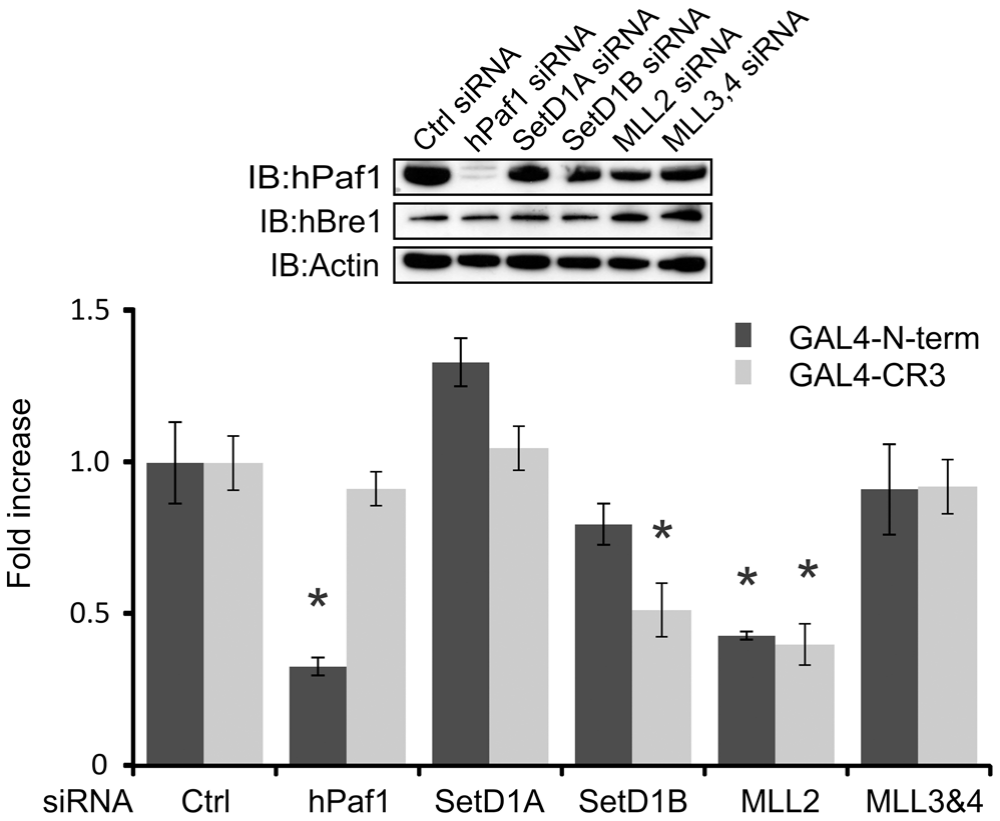
The N-terminus of E1A, but not CR3 requires hPaf1 for efficient transcriptional activation. U-2 OS cells were transfected with a negative Ctrl siRNA or siRNAs specific for known hBre1 interacting proteins. Cells were then transfected with a constitutive β-galactosidase reporter, a Gal4 responsive luciferase reporter and a vector expressing the Gal4 DBD alone, Gal4 DBD fused to the N-terminus of E1A, or Gal4 DBD fused to E1A CR3. Luciferase activity was measured. Results were normalized to β-galactosidase activity and siRNA treated groups were set as a fold to the Gal4 only transfected counterpart. A statistically significant decrease from Ctrl siRNA treatment is indicated (* P<0.01). n = 3.

In addition to its function in H2B monoubiquitination, hBre1 interacts with several other complexes involved in transcriptional regulation [24], [25], [30], [31]. To determine if any of these hBre1 interacting complexes were similarly required for E1A dependent transcriptional activation, U-2 OS cells were treated with siRNA specific to hPaf1, SetD (SET domain containing) 1A, SetD1B, MLL (myeloid/lymphoid or mixed-lineage leukemia) 2, or MLL3 & 4. Cells were then transfected with a constitutive β-galactosidase reporter, a Gal4 responsive luciferase reporter and a vector expressing Gal4 or Gal4 fused to the N-terminus of E1A. Gal4 fused to E1A CR3 was again used as a control, as it functions as a strong transactivator, but does not bind to hBre1. Knockdown of hPaf1 did not affect hBre1 expression (Figure 4, inset), but nevertheless resulted in significant decreases in transcriptional activation by the N-terminus of E1A, but not CR3 (Figure 4). This result is similar to what was observed for knockdown of hBre1 (Figure 1) and suggests that the hPaf1 complex is specifically recruited by the N-terminus of E1A to enhance transcriptional activation. None of the other knockdowns specifically reduced activation by the N-terminus of E1A as they also reduced activation by CR3, which does not bind to hBre1 (Figure 4).

### hPaf1 is recruited to the viral E3 and E4 gene promoters in an E1A and hBre1 dependent manner

**Figure 5:**
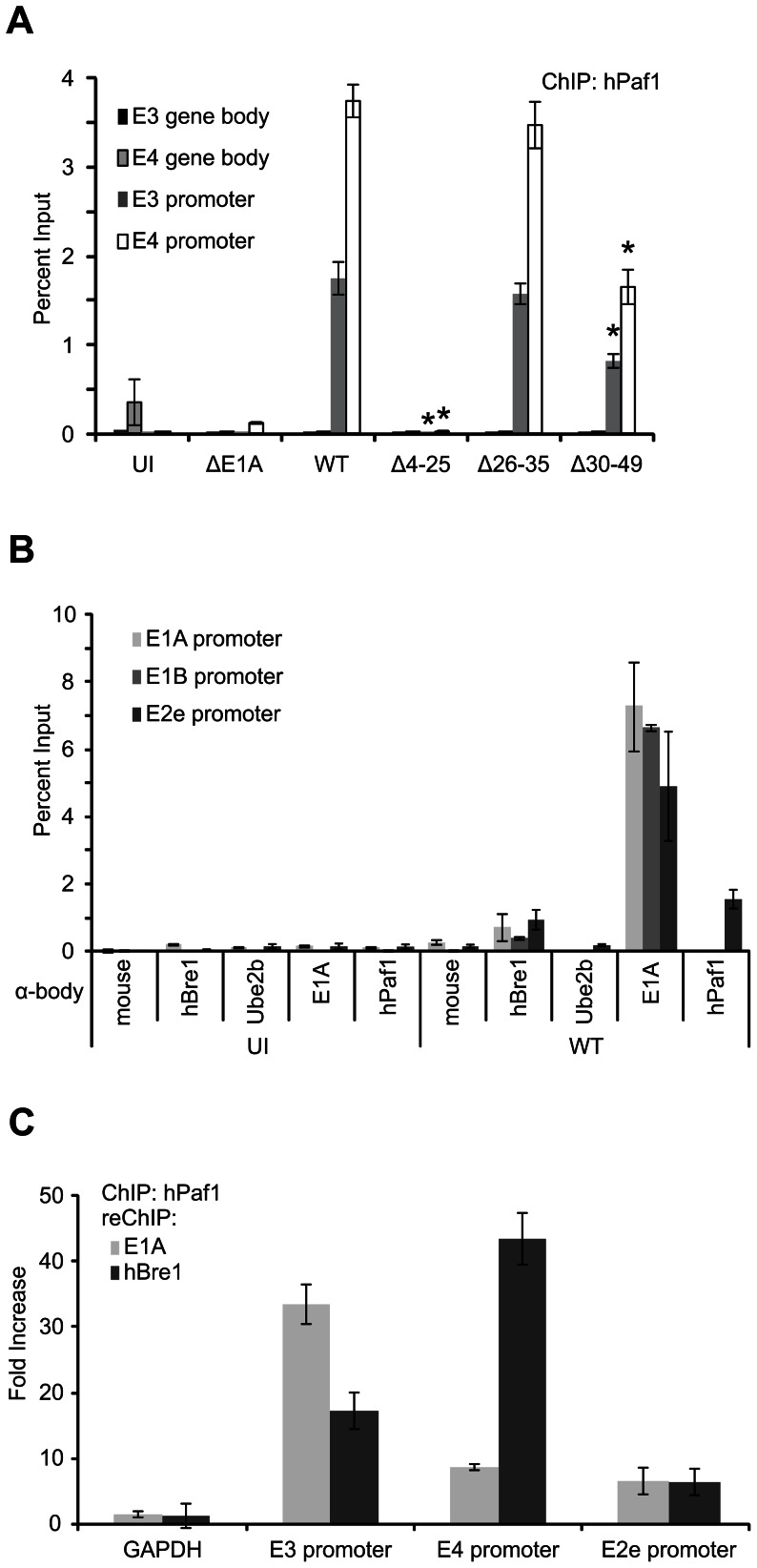
E1A recruits hPaf1 to HAdV early gene promoters via hBre1. (A, B) hPaf1 is localized at the E2e, E3 and E4 promoters. A549 cells were infected with WT HAdV, ΔE1A HAdV or HAdV containing deletions within E1A for 20 hours. Chromatin immunoprecipitation (ChIP) was performed with the indicated antibodies and DNA was probed via qRT-PCR for the presence of HAdV early gene promoters or transcribed regions. hPaf1 localization requires residues 4–25 of E1A and s found on the E2e, E3 and E4 but not the E1A or E1B promoter regions. A statistically significant decrease from WT in A is indicated (* P<0.01). (C) hPaf1 colocalizes with E1A and hBre1 on the E3 and E4 promoters. hPaf1 ChIP was followed by re-ChIP with either hBre1 or E1A specific antibodies to determine co-occupancy. Data was normalized to ΔE1A HAdV and a non-specific antibody control. (D and E) hPaf1 recruitment to the E3 and E4 promoter requires hBre1. A549 cells were treated with a non-specific siRNA, or siRNA specific for hBre1 (D), or hPaf1 (E) prior to virus infection. ChIP assays were then performed using hBre1 or hPaf1 specific antibodies. hPaf1 and hBre1 occupancy was then determined at the HAdV E3 and E4 promoters as described above. n = 3.

To determine whether hPaf1 is recruited to the HAdV genome, we performed ChIP analysis. A549 cells were infected with WT HAdV and a series of HAdV containing deletions in E1A at an MOI of 5. ChIP was then performed using an antibody control and a hPaf1 specific antibody. hPaf1 was found to specifically localize to the E2e, E3 and E4 viral early gene promoters in an E1A dependent manner, and this required the same region of E1A (residues 4–25) necessary for interaction with hBre1 (Figure 5A and B). Reduced hPaf1 occupancy of the E3 and E4 promoters was also observed during infection with virus expressing E1A lacking residues 30–49 of E1A. Although this reduction was not as substantial as with the Δ4–25 mutant, the Δ30–49 mutant retains binding to hBre1, suggesting that other factors such as p300/CBP may influence hPaf1 recruitment. ChIP analysis did not detect hPaf1 within the E3 and E4 gene transcribed regions (Figure 5A), the E1A and E1B promoters (Figure 5B), or IFN responsive genes (Figure S3 in Text S1) during infection suggesting a specific recruitment and localization to the E2e, E3 and E4 promoters. Furthermore, ChIP reChIP experiments demonstrated that hPaf1 co-occupied the E2e, E3 and E4 promoter regions with both hBre1 and E1A (Figure 5C) but did not show any co-localization with either hBre1 or E1A at E1A or E1B promoters (Figure S4A in Text S1). This colocalization on HAdV early genes suggests that E1A, by binding hBre1, is recruiting hPaf1 to participate in the transcriptional activation of the viral E3 and E4 early genes.

Next, we tested whether hPaf1 recruitment to HAdV early genes required hBre1. A549 cells were treated with either a non-specific control siRNA or an siRNA specific to hBre1 and infected with WT HAdV at an MOI of 5. ChIP was then performed using an antibody control or an antibody specific to hPaf1. Knockdown of hBre1 substantially affected hPaf1 recruitment to the E2e, E3 and E4 promoters during HAdV infection, but not E1A, E1B or GAPDH (Figure S4B in Text S1). In contrast, siRNA knockdown of hPaf1 did not reduce hBre1 recruitment to the HAdV genome during HAdV infection (Figure S4B in Text S1). These results indicate that E1A is utilizing hBre1 as a scaffold to recruit hPaf1 to the E3 and E4 promoters. Further, hPaf1 was shown to interact with E1A by co-immunoprecipitation. This interaction was dependent on hBre1 expression, as knockdown of hBre1 reduced hPaf1 co-association with E1A (Figure S4C in Text S1).

### hPaf1 is required for expression of the viral E3 and E4 transcription units

**Figure 6:**
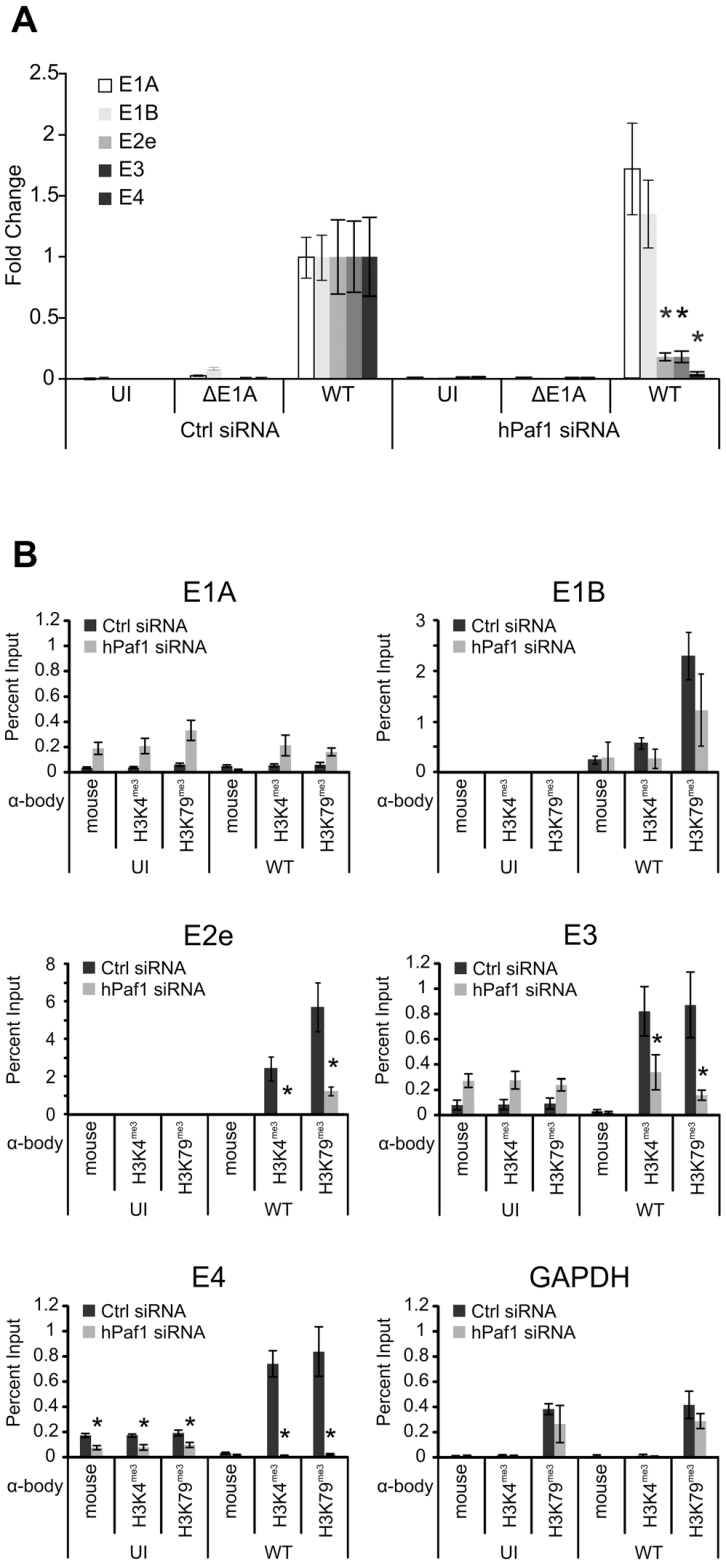
hPaf1 is required for expression of the HAdV E2e, E3 and E4 genes during infection and chromatin marks associated with active gene expression. (A) Knockdown of hPaf1 does not affect E1A and E1B expression, but greatly reduces E3 and E4 expression during infection. A549 cells were treated with Ctrl siRNA or hPaf1 specific siRNA and infected with wildtype (WT) or ΔE1A HAdV. qRT-PCR was then performed for E3 and E4 expression. Data was normalized to GAPDH and fold was set to the WT CTRL siRNA of the same gene. (B) hPaf1 is required for H3K4 and H3K79 trimethylation at the E3 and E4 promoters. ChIP assays were performed on A549 cells treated with siRNA as indicated and infected for 20 hours at a MOI of 5. ChIP was then performed using antibodies specific for trimethylated H3K4 or H3K79 and qRT-PCR was performed for the indicated HAdV early gene promoter region. A statistically significant decrease from Ctrl siRNA treatment is indicated (* P<0.01). n = 3.

To determine if hPaf1 is involved in the transcriptional activation of HAdV early genes, A549 cells were treated with control or hPaf1 specific siRNAs and infected with WT or ΔE1A virus. Knockdown of hPaf1 compared to control siRNA treatment caused a reduction in the ability of WT virus to activate expression of the viral E2e, E3 and E4 (Figure 6A), but not the E1A and E1B early genes during infection (Figure 6A). Thus, hPaf1 knockdown affected the expression of same subset of early genes affected by knockdown of hBre1 (Figure 2). These data confirm that hPaf1 is involved in E1A dependent activation of viral E2e, E3 and E4 gene expression.

### Recruitment of hPaf1 is required for H3K4 and H3K79 tri-methylation of the HAdV E3 and E4 promoters

Recruitment of the hPaf1 complex to a transcriptional template has been reported to be necessary for several histone post-translational modifications associated with active transcription, including trimethylation of H3K4 and H3K79 [21], [32]. To determine if hPaf1 is required for the observed E1A dependent increase in H3K4 and H3K79 tri-methylation at the viral E3 and E4 promoters, A549 cells were infected as before with WT or ΔE1A virus and ChIP was performed using antibodies specific for H3K4 or H3K79 trimethylation. Knockdown of hPaf1 significantly reduced H3K4 and H3K79 trimethylation at the viral E2e, E3 and E4 promoters, but not the E1A, E1B or GAPDH promoters (Figure 6B). This suggests that hPaf1 plays an essential role in generating these two histone modifications at the E3 and E4 promoters, but not the E1A promoter.

### The E1A proteins from multiple HAdV types target hBre1

**Figure 7:**
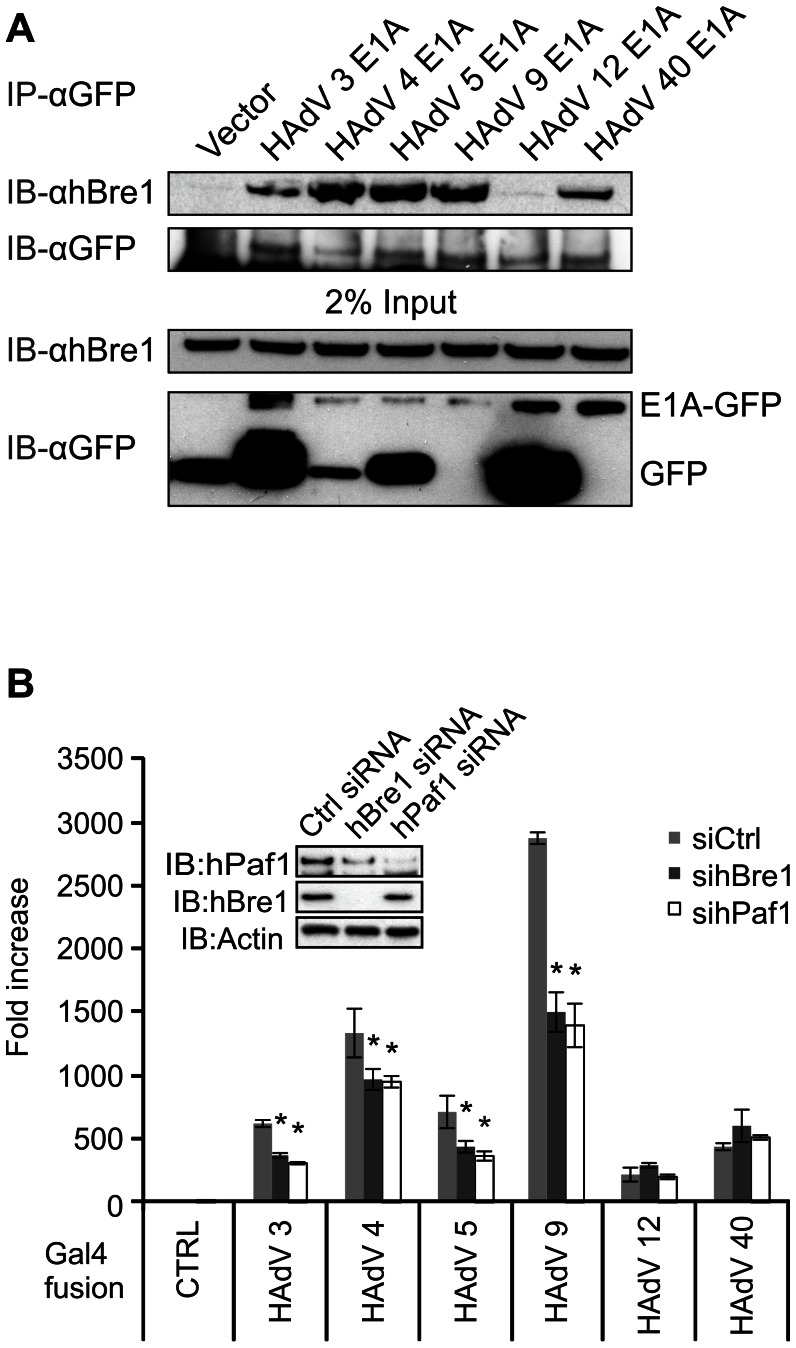
hBre1 is a conserved target of the E1A proteins of multiple types of HAdV. (A) Evolutionary conservation of hBre1 binding with the E1A proteins of multiple HAdV types. HT1080 cells were transfected with hBre1 and either empty control vector, or the E1A genes from representative members of the 6 types of HAdV. E1A was immunoprecipitated and Western blots were performed using hBre1 specific antibody or HA to detect E1A proteins. (B) The E1A proteins from several types utilize hBre1 and hPaf1 for transcriptional activation. U-2 OS cells were treated with Ctrl non-specific siRNA, or an hBre1 or hPaf1 specific siRNA (shown in the inset panel). Cells were then transfected with a constitutive β-galactosidase reporter, a Gal4 responsive luciferase reporter and a vector expressing the Gal4 DBD alone, or Gal4 DBD fused to the N-terminus of the indicated HAdV E1A. Luciferase activity was measured. Results were normalized to β-galactosidase activity and siRNA treated groups were set as a fold to the Gal4 only transfected counterpart. A statistically significant decrease from Ctrl siRNA treatment within the Gal4 fusion group is indicated (* P<0.01). n = 3.

Although it is known that the HAdV 5 E1A protein binds hBre1, it is not known whether this interaction is a feature of all E1A proteins. To determine if the interaction with hBre1 is evolutionarily conserved among the different HAdV types, we co-transfected human HT1080 fibrosarcoma cells with hBre1 and a representative E1A from six different HAdV groups which were tagged at their C-terminus with GFP. Co-immunoprecipitation was performed on lysates by immunoprecipitating the representative E1As with GFP antibody and any co-precipitated hBre1 was detected with hBre1 specific antibody. hBre1 interacted strongly with all of the E1A proteins tested, with the exception of HAdV 12 (Figure 7A). The conservation of the E1A-hBre1 interaction across multiple HAdV species suggests that targeting of hBre1 is an important aspect of E1A function.

### hBre1 and hPaf1 are required for transcriptional activation by the E1A proteins of multiple HAdV types

The evolutionary conservation of the E1A-hBre1 interaction (Figure 7A) suggested that there could also be an evolutionary conservation of E1A-hBre1 function as well. To test this, U-2 OS cells were treated with non-specific siRNA, hBre1 specific siRNA or hPaf1 specific siRNA. Cells were then transfected with a constitutive β-galactosidase reporter, a Gal4 responsive luciferase reporter and a vector expressing the Gal4 DBD or the Gal4 DBD fused to the N-terminus of E1A. The results from the luciferase assays indicate that activation by the N-terminus of the HAdV 3, 4, 5 and 9 E1A proteins types was reduced when either hPaf1 or hBre1 are knocked down (Figure 7B). Thus, the E1A proteins from multiple HAdV types appear to utilize both hBre1 and hPaf1 for transcriptional activation.

## Discussion

**Figure 8:**
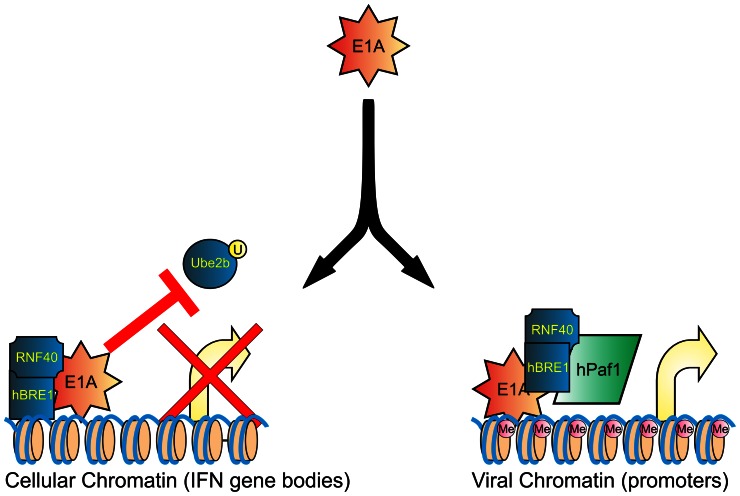
The interaction of E1A with hBre1 serves two completely different purposes during HAdV infection. By disassociating the catalytically active Ube2b component from the hBre1 complex, E1A inhibits H2B monoubiquitination and suppresses transcription of IFN responsive genes (left panel). However, E1A then retasks the catalytically inactive hBre1 complex by using it as a scaffold to recruit hPaf1, leading to localized H3K4 and H3K79 trimethylation and stimulation of viral gene expression.

HAdV E1A is an unusually strong and multifarious regulator of gene expression. E1A is the first protein expressed during viral infection and acts to reprogram cellular gene expression, as well as activate viral early gene expression. As such, E1A serves as a paradigm of eukaryotic transcriptional control. As one particular example, the ability of E1A to bind and sequester the p300/CBP acetyltransferases is a well established mechanism by which E1A can repress cellular transcription [2], [33], [34]. We previously demonstrated that HAdV 5 E1A targets the cellular hBre1 complex. E1A disrupts the interaction between the hBre1 ligase and the Ube2b conjugase, leading to a global reduction in H2B-ub, decreased occupancy by hPaf1 and a consequent abrogation of type I IFN dependent gene expression [20] (Figure S3 in Text S1). The interaction of E1A with hBre1 provides a mechanism by which E1A antagonizes expression of cellular genes required for the innate immune response to viral infection in addition to the sequestration of p300/CBP. In the present work, we have determined that the interaction of E1A with hBre1 is further exploited by the virus to activate expression of the viral E2e, E3 and E4 transcription units. Thus, subversion of the hBre1 complex by E1A results in two distinct and opposite effects: inhibition of transcription from cellular IFN responsive genes and activation of viral gene expression (Figure 8).

Our findings here show a novel means by which hBre1 facilitates transcriptional activation, specifically via recruitment and modulation by the N-terminus of E1A. More importantly, the contribution made by this interaction is completely independent of the known ability of the hBre1 complex to monoubiquitinate H2B. Indeed, neither the Ube2b nor RNF40 components of the hBre1 complex are required for E1A dependent activation of viral early gene expression. This reveals an alternative and unexpected method by which hBre1 can activate transcription. We hypothesized that during infection, E1A functionally retasks the catalytically inactive hBre1 complex, converting it into a scaffold to recruit additional factors that enhance expression of the viral E2e, E3 and E4 early gene promoters. Our results indicate that the outcome of viral retasking of the hBre1 complex is the recruitment of the cellular hPaf1 complex. The hPaf1 complex functions as an important regulator of RNA pol II transcriptional regulation, primarily by promoting transcription elongation and transcription-coupled histone modifications [35]. We demonstrate that hPaf1 was specifically required for transcriptional activation by the N-terminus of HAdV 5 E1A (Figure 4), was recruited to HAdV early genes in an identical pattern to hBre1 and required hBre1 and E1A for this recruitment (Figure 3 and 5). In addition, hPaf1 shared co-occupancy on the viral E2e, E3 and E4 promoters with both E1A and hBre1 and was required for efficient expression of these genes during infection (Figure 5 and 6). In contrast, hPaf1 was not recruited to the viral E1A and E1B promoters and was also not required for their expression (Figure S4B in Text S1, 5B and 6A), suggesting that additional factors are involved in activating expression of these genes.

CR3 is known to be the primary activation domain of viral transcription in HAdV 5 E1A. This is evidenced in that the 289R E1A product activates viral transcription far more strongly than the 243R E1A product, which lacks CR3 [15], [16]. Also, CR3 alone is able to activate viral transcription [10], [15]. However, activation of early gene transcription is greatly enhanced by the presence of the N-terminus of E1A [13], [15]. This functional cooperation may be mediated in part by the co-recruitment of the p300/CBP histone acetyltransferases by the N-terminus and CR3 regions of E1A HAdV 5 [13], [19]. The data presented here suggests that, in addition to p300/CBP, the N-terminus also contributes to viral early gene activation by recruiting hBre1. E1A dependent promoter occupancy by hBre1 in turns recruits the hPaf1 complex and in turn recruits additional factors that further modify viral chromatin, including the appearance of transcriptional activation marks such as H3K4 and H3K79 trimethylation (Figure 6B).

The N-terminus of the E1A proteins from multiple HAdV species interact with hBre1 (Figure 7A). This preservation of E1A interaction suggests a strong evolutionary conservation of functional utility. Knockdown of hBre1 or hPaf1 reduced transactivation by HAdV3, 4 and 9 (Figure 7B), corroborating that their conserved ability to bind hBre1 contributes to transcriptional activation, as we observed with HAdV 5 E1A. Although HAdV 40 E1A bound hBre1, knockdown of hBre1 or hPaf1 did not affect its transactivation ability. Interestingly, HAdV 40 E1A exhibits a deficiency in activating transcription of other early viral genes during infection [36], which could arise from its inability to productively utilize hBre1 to activate transcription. The interaction of HAdV 40 E1A with hBre1 may instead be primarily involved in perturbing the composition of the hBre1 complex to antagonize cellular IFN dependent gene expression, rather than enhancing E1A dependent transcriptional activation. Interestingly, HAdV 12 E1A differs from all the other E1A proteins tested in that it did not bind to hBre1 and similarly showed no requirement for hBre1 or hPaf1 in transcriptional activation (Figure 7B). The inability HAdV 12 E1A to bind hBre1 would be predicted to lead to weaker transactivation, and this may explain why HAdV 12 infection progresses more slowly and yields less virus compared to species C viruses, such as HAdV2/5 [37]. In addition, HAdV 12 infection also differs from species C infection in that it is also less able to evade the type I IFN response [37], which would also be expected based on HAdV 12 E1A's inability to bind hBre1.

The region spanning residues 4–25 on E1A, which is required for interaction with hBre1 [20] and to recruit Paf1 to early gene promoters (Figure 5A), is also required for binding to the p300 and CBP acetyltransferases [26], [27]. Both p300 and CBP are global cellular transcriptional regulators that function through their ability to acetylate proteins including histones and to recruit additional transcriptional regulators [2], [29], [38]–[41]. As mentioned above, p300 and CBP have documented roles in transcriptional regulation of viral early genes [13], [19]. It remains possible that the interaction of E1A with p300/CBP may also assist hBre1 with the recruitment of hPaf1 or function independently to recruit hPaf1. This is supported by a number of our observations. Firstly, knockdown of hBre1 does not completely abrogate hPaf1 recruitment to viral early genes (Figure S4B in Text S1). Secondly, an E1A mutant lacking residues 30–49, which binds to hBre1 but not p300/CBP, also exhibited reduced hPaf1 recruitment to HAdV early genes (Figure 5A). This reduction was not as pronounced as for the Δ4–25 mutant, which binds neither hBre1 nor p300/CBP, supporting roles for both hBre1 and p300/CBP in hPaf1 recruitment. Finally, knockdown of hPaf1 reduced viral early gene transcription to a greater extent than did knockdown of hBre1 (Figure 2 and 6A). Taken together, these data suggest that one function of p300/CBP in early viral gene expression may be to assist hBre1 with the recruitment of hPaf1. This could occur via direct interaction, alterations in chromatin acetylation that influence hPaf1 binding, or acetylation of other non-chromatin factors involved in hPaf1 recruitment. Although both hBre1 and p300/CBP require residues 4–25 for interaction with E1A, p300/CBP also requires residues 36–69 for binding [26], [27]. It remains to be determined if E1A can simultaneously interact with both hBre1 and p300/CBP, as has been shown for p300/CBP and pRb [42], or whether E1A interacts sequentially with these factors to prepare the local chromatin environment for efficient early viral gene expression.

Although no other viral proteins besides E1A have been shown to bind hBre1, several others target hPaf1. Recently, hPaf1 was shown to bind the Influenza A H3N2 NS1 protein [43]. This interaction provides a mechanism by which Influenza may block the IFN response [43], similarly to what we have reported to result from the interaction of E1A with hBre1 [20]. It is interesting that these very different viruses have independently evolved to target the IFN response via these two interlocking epigenetic mechanisms. In addition to Influenza NS1, the HIV gene product Tat also interacts with hPaf1 [43], [44]. Tat recruits the hPaf1 complex along with numerous interacting partners to trimethylate H3K4 and activate viral gene expression from the HIV LTR [44]. This is a very similar function to what we have observed with hPaf1 recruitment by E1A. It remains possible that NS1 may also utilize its interaction with hPaf1 to regulate viral transcription in a manner similar to HAdV E1A and HIV Tat, but this has yet to be confirmed. Correspondingly, the interaction of Tat with hPaf1 may also affect the type I IFN response. Nevertheless, HAdV, Influenza A and HIV have convergently evolved to target the cellular hBre1/hPaf1 complexes as a strategy to regulate transcription during infection. The roles of these two complexes in the transcription process are clearly separable, as removal of hPaf1 dependent methylation patterns has similar effects on both cellular and viral genes, whereas hBre1 dependent monoubiquitination of H2B is required for IFN dependent transcription, but is unnecessary for E1A mediated activation of E2e, E3 and E4 gene expression. How E1A compensates for the lack of active H2B monoubiquitination at the E2e, E3 and E4 transcription units remains unknown, but may be related to differences between HAdV chromatin and normal cellular chromatin.

In summary, we have determined that the interaction of E1A with hBre1 serves two completely different purposes during a HAdV infection (Figure 8). E1A inhibits H2B monoubiquitination and suppresses the type I IFN response by disassociation of hBre1 from Ube2b. However, E1A then retasks the catalytically inactive hBre1 complex by using it as a scaffold to recruit hPaf1 and stimulate viral early gene expression. Achieving an inhibition of innate immunity, while simultaneously repurposing a target to enhance viral transcription, represents an elegant example of the incredible economy of action accomplished by a viral regulatory protein through a single protein interaction.

## Materials and Methods

### Cell lines and plasmids

Human adenocarcinoma A549, human fibrosarcoma HT-1080, and human osteosarcoma U-2 OS cells were grown at 37°C with 5% CO_2_ in DMEM (Multicell) supplemented with 10% fetal bovine serum (Gibco). Plasmids were transfected into HT1080 and U-2 OS cells using Superfect reagent (Qiagen) following the manufacturer's recommendations. Transfection efficiency was typically, 70–80% for HT1080 and 40–50% for U-2 OS cell lines. After 48 hours in culture, transfected cells were used for experimentation. E1A's from the HAdV types were cloned as fusions with GFP located at the C-terminus. All E1A N-terminus and CR3 clones used in luciferase experiments were fused at their N-terminus with the Gal4 DNA binding domain.

### Quantitative RT-PCR

Total RNA was prepared with Trizol extraction (Invitrogen). A total of 1 µg of RNA was reverse transcribed into cDNA by random priming using the One step RT-PCR kit (Qiagen) following the manufacturers' instructions. Quantification of cDNA was done using SYBR-Green Supermix for real-time qPCR (MyIQ, BioRAD) with oligonucleotide sequences that specifically recognize the indicated target. GAPDH was used as a control for total cDNA. Controls without reverse transcriptase were done for each RNA sample alongside the cDNA control. Results were normalized to the GAPDH and uninfected sample. The oligonucleotide sequences are listed in Table S1 in Text S1.

### RNAi knockdown

Downregulation of hBre1, RNF40, and hBre1 interacting proteins, including hPaf1 was performed using Silencer Select siRNA (Ambion). siRNA was delivered to cells via transfection with Silentfect (BioRad) following the manufacturer's instructions: 3 hours after seeding cells, for a period of 48 hours. A scrambled siRNA was used as a control.

### Virus infection of cells

All viruses are derived from the HAdV 5 dl309 background [45] and express both the larger 289Rand smaller 243R E1A proteins [26]. Cells were infected with WT (dl309), or a panel of HAdV containing the indicated E1A deletion mutations: ΔE1A (dl312), E1A Δ4–25 (dl1101), E1A Δ26–35 (dl1102), E1A Δ30–49 (dl1103). HAdV was used at a multiplicity of infection (MOI) of 5 pfu/cell. Cell cultures were infected at 50% confluence and left for 20 hours. Virus infection was found to be near 100% by fluorescence microscopy under these conditions using virus expressing GFP. Subconfluent cells were collected for further experimentation.

### Gal4 luciferase assay

Cells were transfected with a β-galactosidase reporter plasmid for normalization, a plasmid containing the luciferase gene driven by a 6×Gal4 binding sequence, and the indicated Gal4 DNA binding domain fusion plasmids. After 48 hours, cells were lysed in 200 µL using the supplied lysis buffer (Promega E397A). For detection of luciferase production, 50 µL of lysate was mixed with 50 uL of Luciferase Substrate (Promega E151A) immediately before detection of light as measured using a Berthold Lumat LB 9507. Results were then set to an empty plasmid control and further normalized via β-galactosidase activity as detected by ONPG (Bioshop) assays.

### Western blotting and co-immunoprecipitation

Cells were lysed with NP40 lysis buffer (150 mM NaCl, 50 mM Tris-HCl pH 7.5, 0.1% NP-40) and protein concentrations were determined with BioRad protein assay reagent using BSA as a standard. 0.5 mg of protein lysate was immunoprecipitated with 1 µg of GFP rabbit polyclonal (Table S2 in Text S1) at 4°C for 4 hours. 25 µg of protein was kept as 5% input, except as noted in the figure legend of individual blots. After 3 washes in NP40 lysis buffer, complexes were boiled in 25 µL of sample buffer for 5 min. Proteins were separated on NuPage 4–12% Bis-Tris gradient gels (Invitrogen) and transferred onto a nitrocellulose membrane (Amersham). Membranes were blocked in TBS with 0.1% Tween-20 and 5% skim milk or BSA and blotted with the indicated primary overnight at 4°C. Details for the primary antibodies may be found in Table S2 in Text S1. Horseradish peroxidase conjugated secondary antibodies were detected using ECL Plus western blotting detection system (Amersham).

### Chromatin immunoprecipitation (ChIP) and ChIP-reChIP assays

Approximately 10^7^ cells per sample were cross-linked with 1% formaldehyde at room temperature for 10 min. Cells were washed twice with ice cold PBS and harvested. Cell pellets were lysed in 1 mL of cell lysis buffer (50 mM Tris-HCl [pH 8.1], 10 mM EDTA, 1% SDS, and a protease inhibitor cocktail (Sigma) on ice for 10 min. Lysates were sonicated in an ultrasonic biorupter bath (Diogenode XL-2006 TO) to yield DNA fragment sizes of 200–500 base pairs. Samples were then centrifuged at 10,000×g for 10 min. 1 mg of protein was used for ChIP, 100 µg of this was kept as 5% input. Supernatants were then diluted 10-fold in dilution buffer (20 mM Tris-HCl [pH 8.1], 1% Triton X-100, 2 mM EDTA, 150 mM NaCl and protease inhibitors) and precleared with 50 µL of ChIP protein-A Sepharose (50% slurry of protein A-Sepharose containing 2.5 µg of salmon sperm DNA and BSA/mL) for 50 min at 4°C. Immunoprecipitations were performed overnight at 4°C using 5 µg of the indicated antibody found in Table S2 in Text S1. The next morning, 50 µL of ChIP protein-A Sepharose was incubated with each sample for 2 hrs. Beads were then washed once each with 500 µL of wash buffer 1 (0.1% SDS, 1% Triton X-100, 2 mM EDTA, 20 mM Tris-HCl pH 8.1, 150 mM NaCl), wash buffer II (0.1% SDS, 1% Triton X-100, 2 mM EDTA, 20 mM Tris-HCl pH 8.1, 500 mM NaCl), and wash buffer III (0.25 M LiCl, 1% NP-40, 1% Na-deoxycholate, 1 mM EDTA, 10 mM Tris-HCl pH 8.0) respectively and then washed twice with Tris-EDTA buffer. Immunocomplexes were extracted twice with 150 µL of elution buffer (1% SDS, 0.1 M NaHCO_3_). For ChIP-reChIP, samples were then rediluted 10× with dilution buffer and immunoprecipitation was repeated with a second antibody as indicated. After final elution, 12 µL of 5 M NaCl was added to the 300 µL pooled elution and incubated at 65°C overnight to decrosslink the complexes. DNA was then purified using Quigen PCR Purification Spin Columns. Conditions for qRT PCR using SYBR Green were as per the manufacturers' directions. Briefly, each 15 µL reaction contained 80 nM oligos and 0.5 uL of ChIP DNA.

### Statistical analysis

All experiments were carried out in duplicate with three replicates, with the exception of those shown in Figure S1 and Figure S4 in Text S1. Figure S1 and Figure S4 in Text S1 were carried out in duplicate with two replicates. Graphs represent the mean and standard error of the mean (S.E.M) of all experiments, while western blots are representative experiments. All numerical values represent means ± S.E.M. For blots, a representative example of the replicates is shown. Statistical significance of the differences was calculated using one way ANOVA and a Holm-Sidak post-hoc comparison to all other treatments in the experiment.

## Supporting Information

Text S1 Supplementary materials including Figure S1, S2, S3, S4 and Table S1 & S2
(PDF)

## References

[1] Frisch SM, Mymryk JS (2002) Adenovirus-5 E1A: paradox and paradigm. Nature Reviews Molecular Cell Biology 3: 441–452. 12042766 10.1038/nrm827

[2] Ferrari R, Berk AJ, Kurdistani SK (2009) Viral manipulation of the host epigenome for oncogenic transformation. Nature reviews Genetics 10: 290–294. 10.1038/nrg2539 PMC2692573 19290008

[3] Hearing P, Shenk T (1986) The adenovirus type 5 E1A enhancer contains two functionally distinct domains: one is specific for E1A and the other modulates all early units in cis. Cell 45: 229–236. 2938742 10.1016/0092-8674(86)90387-9

[4] Avvakumov N, Wheeler R, D'Halluin JC, Mymryk JS (2002) Comparative Sequence Analysis of the Largest E1A Proteins of Human and Simian Adenoviruses. Journal of Virology 76: 7968–7975. 12134001 10.1128/JVI.76.16.7968-7975.2002 PMC155151

[5] Pelka P, Ablack JNG, Fonseca GJ, Yousef AF, Mymryk JS (2008) Intrinsic structural disorder in adenovirus E1A: a viral molecular hub linking multiple diverse processes. Journal of Virology 82: 7252–7263. 18385237 10.1128/JVI.00104-08 PMC2493305

[6] Geisberg JV, Lee WS, Berk AJ, Ricciardi RP (1994) The zinc finger region of the adenovirus E1A transactivating domain complexes with the TATA box binding protein. Proceedings of the National Academy of Sciences of the United States of America 91: 2488–2492. 8146144 10.1073/pnas.91.7.2488 PMC43394

[7] Yousef AF, Brandl CJ, Mymryk JS (2009) Requirements for E1A dependent transcription in the yeast Saccharomyces cerevisiae. BMC molecular biology 10: 32. 19374760 10.1186/1471-2199-10-32 PMC2674444

[8] Bondesson M, Mannervik M, Akusjärvi G, Svensson C (1994) An adenovirus E1A transcriptional repressor domain functions as an activator when tethered to a promoter. Nucleic acids research 22: 3053–3060. 8065919 10.1093/nar/22.15.3053 PMC310275

[9] Stevens JL, Cantin GT, Wang G, Shevchenko A, Shevchenko A, et al (2002) Transcription control by E1A and MAP kinase pathway via Sur2 mediator subunit. Science 296: 755–758. 11934987 10.1126/science.1068943

[10] Mazzarelli JM, Mengus G, Davidson I, Ricciardi RP (1997) The transactivation domain of adenovirus E1A interacts with the C terminus of human TAF(II)135. Journal of Virology 71: 7978–7983. 9311891 10.1128/jvi.71.10.7978-7983.1997 PMC192158

[11] Grand RJ, Turnell AS, Mason GG, Wang W, Milner AE, et al (1999) Adenovirus early region 1A protein binds to mammalian SUG1-a regulatory component of the proteasome. Oncogene 18: 449–458. 9927201 10.1038/sj.onc.1202304

[12] Duyndam MC, Van Dam H, Van Der Eb AJ, Zantema A (1996) The CR1 and CR3 domains of the adenovirus type 5 E1A proteins can independently mediate activation of ATF-2. Journal of Virology 70: 5852–5859. 8709204 10.1128/jvi.70.9.5852-5859.1996 PMC190602

[13] Wong HK, Ziff EB (1994) Complementary functions of E1a conserved region 1 cooperate with conserved region 3 to activate adenovirus serotype 5 early promoters. Journal of Virology 68: 4910–4920. 8035489 10.1128/jvi.68.8.4910-4920.1994 PMC236431

[14] Ablack JNG, Cohen M, Thillainadesan G, Fonseca GJ, Pelka P, et al (2012) Cellular GCN5 is a novel regulator of human adenovirus E1A-conserved region 3 transactivation. Journal of virology 86: 8198–8209. 22623781 10.1128/JVI.00289-12 PMC3421684

[15] Ablack JNG, Pelka P, Yousef AF, Turnell AS, Grand RJA, et al (2010) Comparison of E1A CR3-dependent transcriptional activation across six different human adenovirus subgroups. Journal of Virology 84: 12771–12781. 20881041 10.1128/JVI.01243-10 PMC3004344

[16] Berk AJ (2005) Recent lessons in gene expression, cell cycle control, and cell biology from adenovirus. Oncogene 24: 7673–7685. 16299528 10.1038/sj.onc.1209040

[17] Pelka P, Ablack JNG, Shuen M, Yousef AF, Rasti M, et al (2009) Identification of a second independent binding site for the pCAF acetyltransferase in adenovirus E1A. Virology 391: 90–98. 19541337 10.1016/j.virol.2009.05.024

[18] Hateboer G, Gennissen A, Ramos YF, Kerkhoven RM, Sonntag-Buck V, et al (1995) BS69, a novel adenovirus E1A-associated protein that inhibits E1A transactivation. the The European Molecular Biology Organization Journal 14: 3159–3169. 10.1002/j.1460-2075.1995.tb07318.x PMC394377 7621829

[19] Pelka P, Ablack JNG, Torchia J, Turnell AS, Grand RJA, et al (2009) Transcriptional control by adenovirus E1A conserved region 3 via p300/CBP. Nucleic acids research 37: 1095–1106. 19129215 10.1093/nar/gkn1057 PMC2651774

[20] Fonseca GJ, Thillainadesan G, Yousef AF, Ablack JN, Mossman KL, et al (2012) Adenovirus evasion of interferon-mediated innate immunity by direct antagonism of a cellular histone posttranslational modification. Cell host & microbe 11: 597–606. 22704620 10.1016/j.chom.2012.05.005

[21] Kim J, Guermah M, McGinty RK, Lee J-S, Tang Z, et al (2009) RAD6-Mediated transcription-coupled H2B ubiquitylation directly stimulates H3K4 methylation in human cells. Cell 137: 459–471. 19410543 10.1016/j.cell.2009.02.027 PMC2678028

[22] Osley MA (2004) H2B ubiquitylation: the end is in sight. Biochimica et biophysica acta 1677: 74–78. 15020048 10.1016/j.bbaexp.2003.10.013

[23] Shema E, Tirosh I, Aylon Y, Huang J, Ye C, et al (2008) The histone H2B-specific ubiquitin ligase RNF20/hBRE1 acts as a putative tumor suppressor through selective regulation of gene expression. Genes & development 22: 2664–2676. 18832071 10.1101/gad.1703008 PMC2559905

[24] Kim J, Roeder RG (2009) Direct Bre1-Paf1 Complex Interactions and RING Finger-independent Bre1-Rad6 Interactions Mediate Histone H2B Ubiquitylation in Yeast. The Journal of Biological Chemistry 284: 20582–20592. 19531475 10.1074/jbc.M109.017442 PMC2742823

[25] Wood A, Schneider J, Dover J, Johnston M, Shilatifard A (2003) The Paf1 complex is essential for histone monoubiquitination by the Rad6-Bre1 complex, which signals for histone methylation by COMPASS and Dot1p. The Journal of Biological Chemistry 278: 34739–34742. 12876294 10.1074/jbc.C300269200

[26] Egan CAY, Jelsma TN, Howe JA, Bayley ST, Ferguson B, et al (1988) Mapping of Cellular Protein-Binding Sites on the Products of Early-Region lA of Human Adenovirus Type 5. Molecular and Cellular Biology 8: 3955–3959. 2975755 10.1128/mcb.8.9.3955-3959.1988 PMC365458

[27] Mymryk JS, Lee RW, Bayley ST (1992) Ability of adenovirus 5 E1A proteins to suppress differentiation of BC3H1 myoblasts correlates with their binding to a 300 kDa cellular protein. Molecular biology of the cell 3: 1107–1115. 1421568 10.1091/mbc.3.10.1107 PMC275675

[28] Rasti M, Grand RJA, Yousef AF, Shuen M, Mymryk JS, et al (2006) Roles for APIS and the 20S proteasome in adenovirus E1A-dependent transcription. The EMBO journal 25: 2710–2722. 16763564 10.1038/sj.emboj.7601169 PMC1500861

[29] Ferrari R, Pellegrini M, Horwitz Ga, Xie W, Berk AJ, et al (2008) Epigenetic reprogramming by adenovirus e1a. Science 321: 1086–1088. 18719284 10.1126/science.1155546 PMC2693122

[30] Lee J-S, Shukla A, Schneider J, Swanson SK, Washburn MP, et al (2007) Histone crosstalk between H2B monoubiquitination and H3 methylation mediated by COMPASS. Cell 131: 1084–1096. 18083099 10.1016/j.cell.2007.09.046

[31] Hahn MA, Dickson K-A, Jackson S, Clarkson A, Gill AJ, et al (2012) The tumor suppressor CDC73 interacts with the ring finger proteins RNF20 and RNF40 and is required for the maintenance of histone 2B monoubiquitination. Human molecular genetics 21: 559–568. 22021426 10.1093/hmg/ddr490

[32] Mohan M, Herz H, Takahashi Y, Lin C, Lai KC, et al (2010) Linking H3K79 trimethylation to Wnt signaling through a novel Dot1-containing complex ( DotCom ). Genes & Development 24: 574–589. 20203130 10.1101/gad.1898410 PMC2841335

[33] Jelsma TN, Howe JA, Mymryk JS, Evelegh CM, Cunniff NF, et al (1989) Sequences in E1A proteins of human adenovirus 5 required for cell transformation, repression of a transcriptional enhancer, and induction of proliferating cell nuclear antigen. Virology 171: 120–130. 2568030 10.1016/0042-6822(89)90518-7

[34] Horwitz Ga, Zhang K, McBrian Ma, Grunstein M, Kurdistani SK, et al (2008) Adenovirus small e1a alters global patterns of histone modification. Science 321: 1084–1085. 18719283 10.1126/science.1155544 PMC2756290

[35] Tomson BN, Arndt KM (2012) The many roles of the conserved eukaryotic Paf1 complex in regulating transcription, histone modifications, and disease states. Biochimica et biophysica acta 1829 1: 116–26. 22982193 10.1016/j.bbagrm.2012.08.011 PMC3541448

[36] Van Loon AE, Gilardi P, Perricaudet M, Rozijn TH, Sussenbach JS (1987) Transcriptional activation by the E1A regions of adenovirus types 40 and 41. Virology 160: 305–307. 2957850 10.1016/0042-6822(87)90080-8

[37] Zhao H, Boije H, Granberg F, Pettersson U, Svensson C (2009) Activation of the interferon-induced STAT pathway during an adenovirus type 12 infection. Virology 392: 186–195. 19665745 10.1016/j.virol.2009.07.006

[38] Eckner R, Ewen ME, Newsome D, Gerdes M, DeCaprio JA, et al (1994) Molecular cloning and functional analysis of the adenovirus E1A-associated 300-kD protein (p300) reveals a protein with properties of a transcriptional adaptor. Genes & development 8: 869–884. 7523245 10.1101/gad.8.8.869

[39] Horwitz GA, Zhang K, McBrian MA, Grunstein M, Kurdistani SK, et al (2008) Adenovirus small e1a alters global patterns of histone modification. Science (New York, NY) 321: 1084–1085. 10.1126/science.1155544 PMC2756290 18719283

[40] Chen J, Li Q (2011) Life and death of transcriptional co-activator p300. Epigenetics: official journal of the DNA Methylation Society 6: 957–961. 10.4161/epi.6.8.16065 21730760

[41] Bedford DC, Kasper LH, Fukuyama T, Brindle PK (2010) Target gene context influences the transcriptional requirement for the KAT3 family of CBP and p300 histone acetyltransferases. Epigenetics: official journal of the DNA Methylation Society 5: 9–15. 10.4161/epi.5.1.10449 PMC2829352 20110770

[42] Ferreon JC, Martinez-Yamout Ma, Dyson HJ, Wright PE (2009) Structural basis for subversion of cellular control mechanisms by the adenoviral E1A oncoprotein. Proceedings of the National Academy of Sciences of the United States of America 106: 13260–13265. 19651603 10.1073/pnas.0906770106 PMC2726373

[43] Marazzi I, Ho JSY, Kim J, Manicassamy B, Dewell S, et al (2012) Suppression of the antiviral response by an influenza histone mimic. Nature 483: 428–433. 22419161 10.1038/nature10892 PMC3598589

[44] Sobhian B, Laguette N, Yatim A, Nakamura M, Levy Y, et al (2010) HIV-1 Tat assembles a multifunctional transcription elongation complex and stably associates with the 7SK snRNP. Molecular cell 38: 439–451. 20471949 10.1016/j.molcel.2010.04.012 PMC3595998

[45] Jones N, Shenk T (1979) Isolation of adenovirus type 5 host range deletion mutants defective for transformation of rat embryo cells. Cell 17: 683–689. 476833 10.1016/0092-8674(79)90275-7

