# Supplementary material for: Viral Retasking of hBre1/RNF20 to Recruit hPaf1 for Transcriptional Activation
Source: PLoS Pathog. 2013 Jun 13;9(6):e1003411. doi: 10.1371/journal.ppat.1003411 (PMC3681745; doi:10.1371/journal.ppat.1003411)
Supplement: Text S1 — Supplementary materials including Figure S1, S2, S3, S4 and Table S1 & S2 (PDF) [file ppat.1003411.s001.pdf]

Figure S1

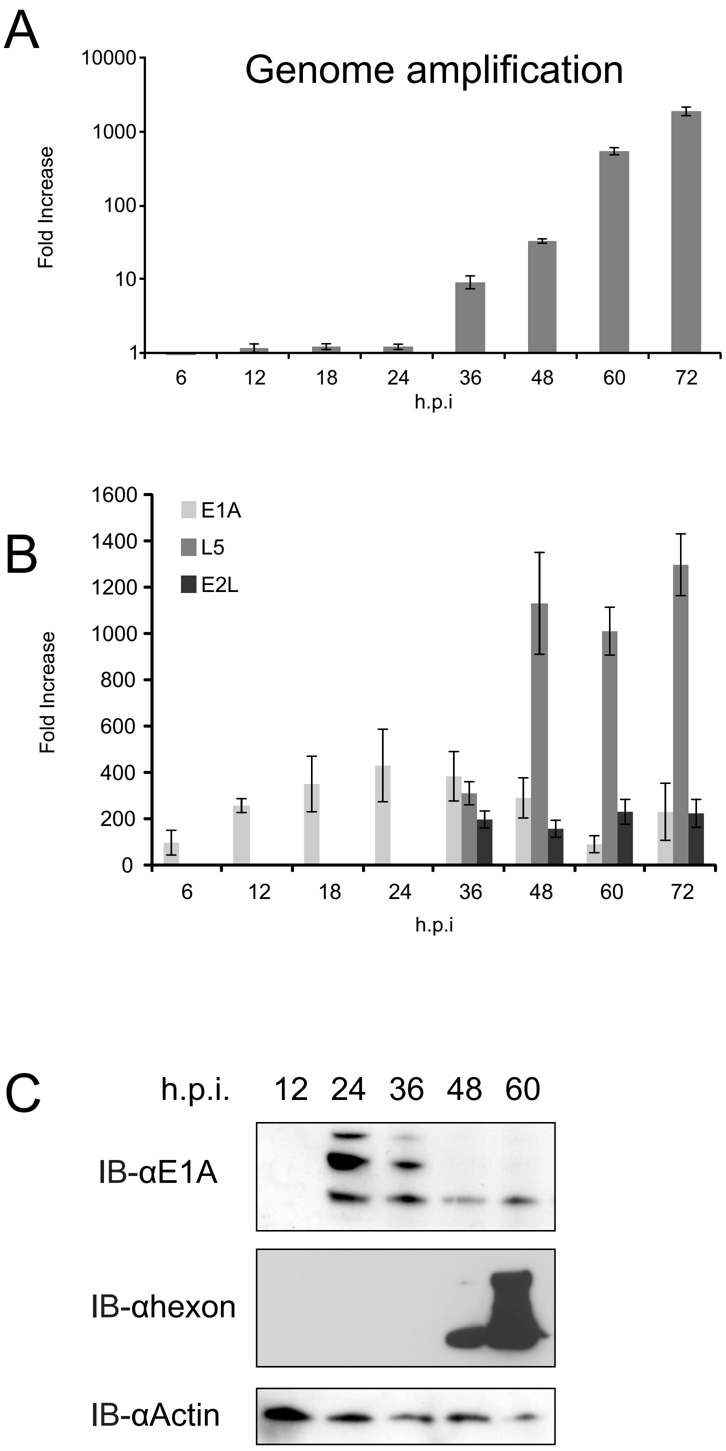
A
Genome amplification
Fold Increase
10000
1000
100
10
1
6 12 18 24 36 48 60 72
h.p.i
B
1600
1400
1200
1000
800
600
400
200
0
E1A
L5
E2L
Fold Increase
6 12 18 24 36 48 60 72
h.p.i
C
h.p.i. 12 24 36 48 60
IB-αE1A
IB-αhexon
IB-αActin

Figure S2

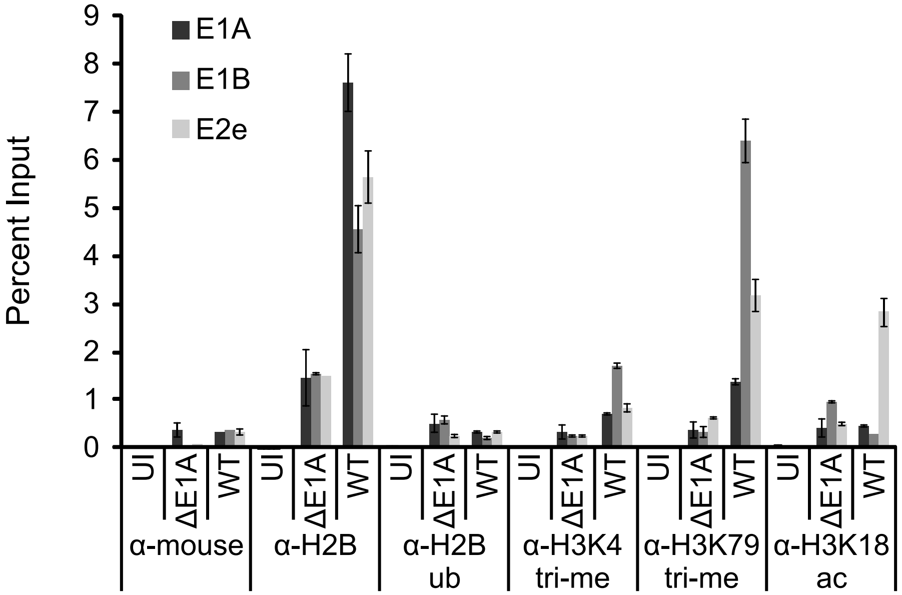
E1A
E1B
E2e
Percent Input
0
1
2
3
4
5
6
7
8
9
UI
ΔE1A
WT
α-mouse
α-H2B
α-H2B ub
α-H3K4 tri-me
α-H3K79 tri-me
α-H3K18 ac

Figure S3

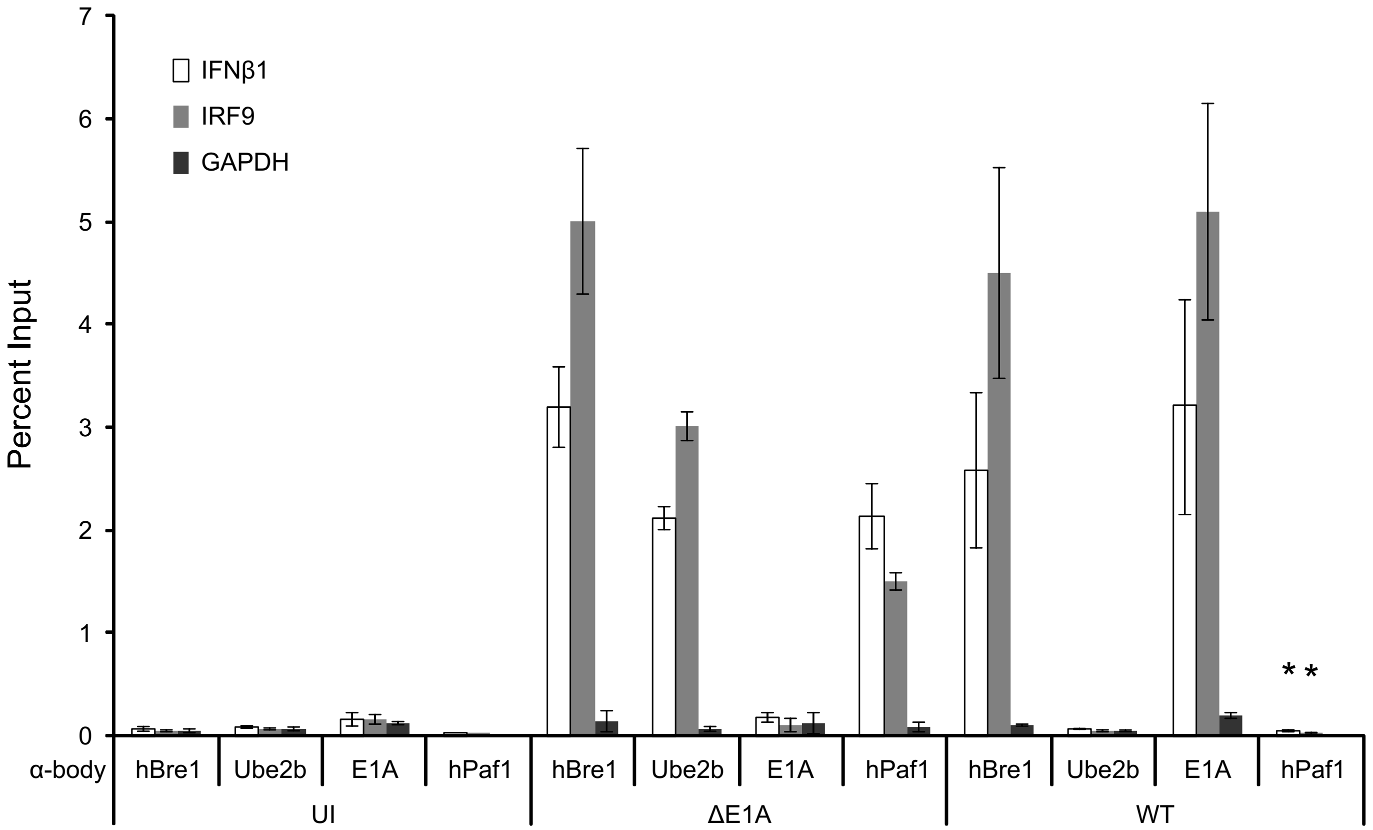

IFNβ1
IRF9
GAPDH
Percent Input
0
1
2
3
4
5
6
7
* *
α-body
hBre1
Ube2b
E1A
hPaf1
hBre1
Ube2b
E1A
hPaf1
hBre1
Ube2b
E1A
hPaf1
UI
ΔE1A
WT

Figure S4

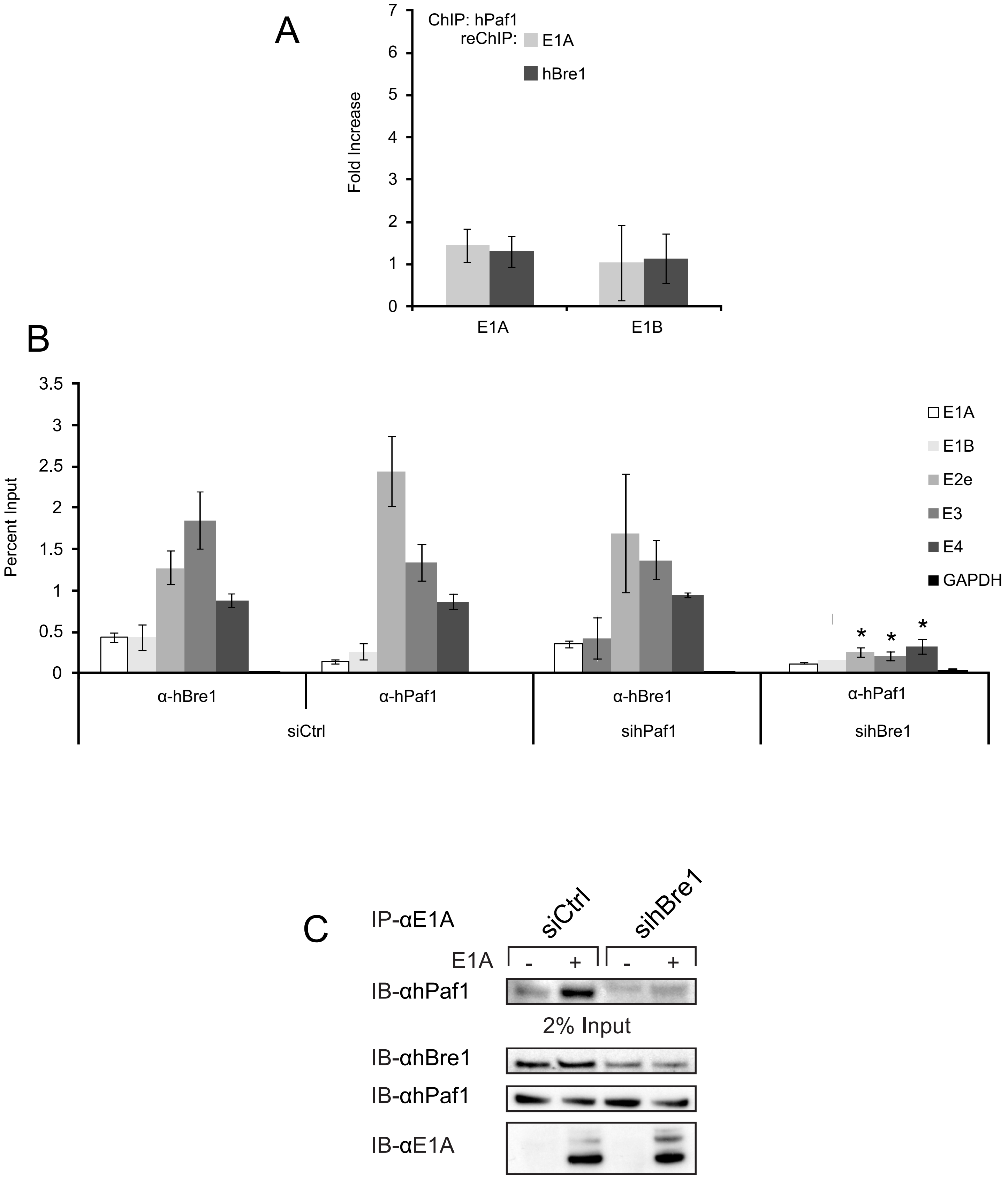
A
ChIP: hPaf1
reChIP:
E1A
hBre1
Fold Increase
7
6
5
4
3
2
1
0
E1A
E1B
B
3.5
3
2.5
2
1.5
1
0.5
0
Percent Input
E1A
E1B
E2e
E3
E4
GAPDH
*
*
*
α-hBre1
α-hPaf1
α-hBre1
α-hPaf1
siCtrl
sihPaf1
sihBre1
C
IP-αE1A
siCtrl
sihBre1
E1A
-
+
-
+
IB-αhPaf1
2% Input
IB-αhBre1
IB-αhPaf1
IB-αE1A

Supplementary Figure Legends

Figure S1. Kinetics of virus infection under the conditions used for all experiments. (A) PCR detection of viral genome amplification. RT-qPCR was performed on DNA collected from the time course of infected cells using primers for the E1A promoter to detect copies of the viral genome and the GAPDH promoter to detect copies of the cellular genome. Results of WT infected cells were normalized to ΔE1A HAdV and set as fold to the cellular genome via the GAPDH promoter. (B) HAdV late gene expression is not detected until 36 h.p.i. A549 cells were infected at an MOI of 5 with WT or ΔE1A HAdV. Cells were collected at the indicated time points and RNA and DNA were collected. RT-qPCR was performed for the HAdV early transcripts E1A and the late genes E2l and L5, and normalized to GAPDH. Fold change to GAPDH was plotted. (C) HAdV late gene protein expression is detected at 48 h.p.i. A549 cells infected with WT virus were collected after 20 h.p.i. and protein was detected via western blotting with the indicated antibodies (E1A represents early gene expression and hexon represents late gene expression). n=2

Figure S2. HAdV early genes E1A, E1B, and E2e display varied hPTM states which do not include H2B-ub association. A549 cells were infected with WT HAdV or ΔE1A HAdV for 20 hours. Chromatin immunoprecipitation (ChIP) was then performed with antibodies specific for the indicated histone or hPTMs. DNA was probed via qRT-PCR for the presence of HAdV early gene promoters E1A, E1B or E2e. Data was normalized to ΔE1A HAdV and a non-specific antibody control. n=3

Figure S3. hPaf1 is excluded from IFN regulated genes during WT HAdV infection. ChIP assays were performed on A549 cells 20 hours after infection with the indicated viruses at a MOI of 5. ChIP was then performed using a control anti-mouse antibody or antibodies specific for hBre1, Ube2b, E1A, or hPaf1. hBre1, Ube2b and hPaf1 associate with IFN regulated gene bodies during IFN stimulating conditions (ΔE1A virus infection). In contrast, in the presence of E1A, Ube2b and hPaf1 are excluded from IFN stimulated gene bodies (WT virus infection). A statistically significant decrease from ΔE1A infected is indicated (* $P<0.01$). n=3

Figure S4. hPaf1 recruitment to the E2e, E3 and E4 promoters requires E1A and hBre1 and the interaction of E1A with hPaf1 requires hBre1. (A) hPaf1 ChIP was followed by re-ChIP with either hBre1 or E1A specific antibodies to determine co-occupancy at the E1A and E1B promoters. Data was normalized to ΔE1A HAdV and a non-specific antibody control. hPaf1 is not co-associated with E1A and hBre1 at these promoters. (B) hPaf1 recruitment to the E2e, E3 and E4 promoters requires hBre1. A549 cells were treated with a non-specific siRNA, or siRNA specific for hBre1, or hPaf1 prior to virus infection. ChIP assays were then performed using hBre1 or hPaf1 specific antibodies. hPaf1 and hBre1 occupancy was then determined at the HAdV E1A, E1B, E2e, E3 and E4 promoters as described above. A statistically significant decrease from Ctrl siRNA treatment is indicated (* $P<0.01$) for B. (C) hPaf1 interacts with E1A and this is hBre1 dependent. A549 cells were treated with Ctrl or hBre1 specific siRNA and infected with WT or ΔE1A virus. Cells were collected after 20 hours. Lysates were immunoprecipitated with anti-E1A antibody and probed for hPaf1. n=2

Table S1. List of primers used in this study.

Target	Region	Location	Forward	Reverse	Size
IFNβ1	Transcript	185-306	AGTGTCAGAAGCTCCTGTGGCAA	TGCGGCGTCCTCCTTCTGGA	122
IRF9	Transcript	902-1010	TTCCCCAAGCCTGGCCCACT	GGCAAAGGCGCTGCACGAAG	109
GAPDH	Transcript	566-644	ACTGCTTAGCACCCCTGGCCAA	ATGGCATGGACTGTGGTCATGAGTC	79
E1A	Transcript	1-207	ATGAGACATATTATCTGC	TTAAGAGTCGGGAAAAATCTG	207
E1B	Transcript	1351-1474	GGTGCAGACCCTGCGAGT	CGCGCTGAGTTTGGCTCTAG	123
E2e	Transcript	17-155	GGGGGTGGTTTCGCGCTGCTCC	GCGGATGAGGCGGCGTATCGAG	138
E2L	Transcript	7345-7474	CTTGGAATACTCAGAGAG	GCCATGCGCGGGCGGCAAGCG	129
E3	Transcript	170-297	AGCTTTCTGAAATGTCCCGTCCGG	CGAGGGCGGCTTTCGTCACAGGG	127
E4	Transcript	1-328	ATGATTCGCTGCTTGAGGCTG	TTATTCCAAAAGATTATCCAAAACCTC	329
L5	Transcript	564-696	GGGCATTGACTTGAAAGAGCCC	TTATTAATAGTCACACCTGG	132
E1A	Promoter	332-527	GCGCGTAATATTTGTCTAGGG	GAAAACTCTACTCGCTGGC	195
E1B	Promoter	1546-1708	GGTGTAAACCTGTGATTGCG	CAGATGTAACCAAGATTAGCCC	162
E2e	Promoter	7918-7793	ACGAGCTGCTTCCCAAAG	ATGGCCTTGATGGACAGC	125
E3	Promoter	27501-27633	TCTGAAATGTCCCGTCCGG	GGCGGCTTTCGTCACAGGG	132
E4	Promoter	35585-35694	CAGCTCAATCAGTCACAGTGTAAAAAGGGCC	TGCGGTTTTCTGGGTGTTTT	110

Table S2. List of antibodies used in this study.

Reactivity	Description	Company	Cat #
Mouse IgG	Rabbit polyclonal	Sigma	M-7023
Actin	Rabbit polyclonal	Sigma	A 2066
GFP	Rabbit polyclonal	Clonetech	632592
hPaf1	Rabbit polyclonal	Bethyl Labs	A300-173A
H2B	Rabbit polyclonal	Millipore	07-371
H2BK120-monoubiquitination	Mouse monoclonal	Millipore	05-1312
H3K4 tri methylation	Mouse monoclonal	Abcam	Ab1012
H3K18 acetylation	Rabbit polyclonal	Abcam	Ab1191
H3K79 tri methylation	Rabbit polyclonal	Abcam	Ab2621
hBre1/RNF20	Mouse monoclonal	Sigma	R8904
M73 (E1A)	Mouse monoclonal	In house	
RNA Pol II	Rabbit polyclonal	Abcam	Ab26721
RNF40/hBre1b/RBP95	Mouse monoclonal	Sigma	R9029
Ube2b/hRad6	Rabbit polyclonal	Bethyl Labs	A300-281A
9C12 (hexon)	Mouse monoclonal	DSHB	TC31-9C12.C9
27F11 (hexon)	Mouse monoclonal	DSHB	TC31-27F11.C2
